# Triglyceride-glucose index and its derivatives as emerging biomarkers of insulin resistance for prognostic evaluation of cardiovascular-kidney-metabolic syndrome: mechanisms and clinical applications

**DOI:** 10.3389/fphar.2026.1787130

**Published:** 2026-03-09

**Authors:** Zhiming Zheng, Xueshi Yin, Jianping Liu, Yongheng Zhang

**Affiliations:** 1 Department of Clinical Medicine, North Sichuan Medical College, Nanchong, Sichuan, China; 2 Department of Cardiac and Great Vascular Surgery, Suining Central Hospital, Suining, Sichuan, China

**Keywords:** biomarker, cardiovascular-kidney-metabolic syndrome, insulin resistance, prognosis, triglyceride-glucose

## Abstract

Cardiovascular-Kidney-Metabolic Syndrome (CKM Syndrome) is a complex, multi-organ disorder characterized by metabolic dysfunction, involving the cardiovascular, renal, and metabolic systems. The components of CKM Syndrome exhibit significant interactions, driving the progression of multi-organ dysfunction. Insulin resistance (IR), a key driver of metabolic disturbances, plays a central role in the pathogenesis of CKM Syndrome. Traditional methods for assessing IR are often invasive and impractical for clinical and large-scale research use. Therefore, novel biomarkers, such as the triglyceride-glucose (TyG) index and its derivatives, have emerged as promising tools for identifying high-risk populations in CKM Syndrome. This review aims to provide a comprehensive analysis of the current application and potential of emerging biomarkers for IR in the prognostic evaluation of CKM. We focus on the clinical pathophysiological mechanisms underlying IR and its role in CKM, including disturbances in glucose and lipid metabolism, inflammatory responses, oxidative stress, endothelial dysfunction, and renal injury. The TyG index, which integrates fasting blood glucose and triglycerides, has shown significant potential in predicting CKM-related outcomes. Furthermore, derivative indices, such as the dynamic TyG and combined TyG index, offer enhanced predictive capability by incorporating additional physiological and metabolic parameters. Despite the promising applications of the TyG index and its derivatives, several limitations exist, including the lack of standardized thresholds, variability across populations, and challenges in dynamic monitoring. Future research should focus on elucidating the molecular mechanisms of IR, conducting large-scale multi-center studies, and developing individualized monitoring models to improve the clinical utility of these biomarkers. The TyG index holds significant potential for early identification, risk stratification, and intervention in CKM, offering a new strategy for improving patient outcomes and transforming chronic disease management.

## 1 Introduction

Cardiovascular-Kidney-Metabolic Syndrome (CKM Syndrome) is a complex, multi-organ syndrome characterized by metabolic dysfunction that involves the interaction between the cardiovascular, renal, and metabolic systems (Ndumele et al., 2023a). The components of CKM exhibit significant interactivity, which collectively contributes to the progression of multi-organ dysfunction. The American Heart Association (AHA) categorizes CKM into five stages, from 0 to 4, with stage 4 representing the most severe clinical manifestation (Table 1) (Ndumele et al., 2023b). Epidemiological data show that approximately 80% of Chinese adults suffer from CKM Syndrome, with 75% of these individuals in the early stages (stages 0–3), and the prevalence continues to rise annually (He et al., 2025). In the United States, about 9% of adults have advanced CKM Syndrome (stage 4, clinical symptom stage) (Aggarwal et al., 2024). The high incidence and rapid progression of CKM result in poor prognoses, making early identification of high-risk individuals critical for improving outcomes.

**TABLE 1:** Definitions of CKM syndrome stages.

Stage	Definition	Criteria
Stage 0	No CKM risk factors	Normal BMI and waist circumference; normal glucose, blood pressure, and lipid profile; no evidence of CKD or clinical/subclinical CVD.
Stage 1	Excess or dysfunctional adiposity (with or without prediabetes)	BMI ≥25 kg/m^2^ (or ≥23 kg/m^2^ for Asian populations); and/or waist circumference ≥88 cm (women) or ≥102 cm (men) (or ≥80/90 cm for Asian women/men). Prediabetes may be present (fasting glucose 100–124 mg/dL or HbA1c 5.7%–6.4%)
Stage 2	Metabolic risk factors and/or CKD	Metabolic risk factors (e.g., hypertriglyceridemia ≥135 mg/dL, hypertension, diabetes, metabolic syndrome) and/or CKD (moderate- to high-risk CKD per guideline-based classification)
Stage 3	Subclinical CVD in the setting of excess/dysfunctional adiposity, metabolic risk factors, or CKD	Subclinical ASCVD (e.g., coronary artery calcium or imaging evidence of atherosclerosis) and/or subclinical HF supported by biomarkers (NT-proBNP ≥125 pg/mL; hs-troponin T ≥ 14 ng/L in women and ≥22 ng/L in men; hs-troponin I ≥ 10 ng/L in women and ≥12 ng/L in men) and/or imaging evidence of structural/functional abnormalities
Stage 4	Clinical CVD (± kidney failure)	Established clinical CVD (e.g., CAD, HF, stroke, PAD, atrial fibrillation). Stage 4a: without kidney failure; Stage 4b: with kidney failure

CKM, staging definitions are adapted from the AHA, Presidential Advisory and Scientific Statement and related contemporary reviews (Ndumele et al., 2023a; Ndumele et al., 2023b; Sebastian et al., 2024).

Biomarkers are crucial tools for identifying high-risk individuals in clinical practice. Insulin resistance (IR), a key driver of metabolic dysfunction, plays a central role in the pathogenesis of CKM Syndrome (Liu K. et al., 2025). However, traditional methods for assessing IR, such as the hyperinsulinemic-euglycemic clamp, are complex, expensive, and invasive, making them unsuitable for widespread use in clinical practice or large-scale studies. Consequently, there is an urgent need for emerging biomarkers that are simple yet well-supported by evidence. Recently, novel biomarkers such as the triglyceride-glucose (TyG) index and its derivatives, with their precise calculation methods and multifactorial integration, have shown great potential in identifying high-risk populations for CKM Syndrome.

At present, CKM Syndrome risk stratification mainly relies on conventional metabolic indicators and established cardio-renal markers, yet these approaches may not sufficiently capture the early systemic metabolic disturbances driven by IR. In this context, the TyG index provides a practical surrogate of IR by integrating fasting glucose and triglycerides, thereby reflecting combined glucotoxicity and lipotoxicity along the CKM Syndrome continuum. However, the clinical translation of TyG-related indices remains challenged by several unresolved issues, including non-standardized cutoff thresholds, heterogeneity across populations (e.g., age, sex, obesity status, diabetes, and renal dysfunction), and increasingly reported non-linear associations with clinical outcomes (e.g., U-shaped or J-shaped relationships). Therefore, this review summarizes the pathophysiological mechanisms linking IR to CKM Syndrome progression, highlights the clinical applications of the TyG index and its derivative indices (including dynamic and combined models) for prognostic evaluation, discusses current limitations and controversies, and proposes future directions toward standardized and implementable IR-based risk assessment strategies in CKM Syndrome.

## 2 Pathophysiological mechanisms

**FIGURE 1:**
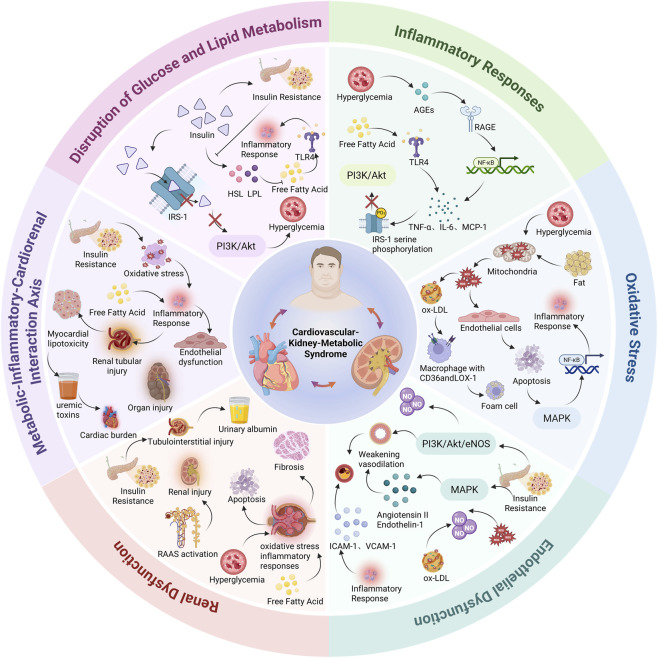
Schematic Representation of Pathophysiological Mechanisms in Cardiovascular-Kidney-Metabolic Syndrome (CKM). This schematic diagram illustrates the complex interplay of six key pathophysiological mechanisms implicated in the development of CKM: disruption of glucose and lipid metabolism, inflammatory responses, oxidative stress, endothelial dysfunction, renal dysfunction, and the metabolic-inflammatory-cardiorenal interaction axis.

Insulin resistance (IR) is a central pathological feature in the development of Cardiovascular-Kidney-Metabolic Syndrome (CKM Syndrome). It not only causes metabolic dysfunction but also activates specific signaling pathways and molecular mechanisms that trigger inflammatory responses, oxidative stress, endothelial dysfunction, and kidney injury. These mechanisms are interlinked and drive the progression of CKM Syndrome toward more severe stages (Figure 1). Importantly, these key pathophysiological disturbances are clinically mirrored by the TyG index, which integrates fasting triglycerides and fasting glucose as a pragmatic surrogate of IR. Under IR, impaired suppression of adipose lipolysis increases circulating free fatty acids and hepatic very-low-density lipoprotein (VLDL) production, elevating triglycerides (lipotoxicity), while reduced peripheral glucose uptake together with enhanced hepatic gluconeogenesis increases fasting glucose (glucotoxicity). By simultaneously capturing dysregulated lipid and glucose metabolism, TyG provides a more robust reflection of the systemic metabolic milieu linked to inflammation, vascular dysfunction, and renal hemodynamic stress than either triglycerides or glucose alone, offering a mechanistically plausible marker for prognostic evaluation within the CKM Syndrome continuum.

### 2.1 Disruption of glucose and lipid metabolism

The direct consequence of IR is impaired insulin signaling, particularly the disruption of insulin receptor substrates (IRS-1) and the phosphatidylinositol 3-kinase (PI3K)/Akt pathway, leading to a reduced ability of peripheral tissues (such as skeletal muscle and adipose tissue) to uptake glucose. At the same time, hepatic gluconeogenesis is activated, increasing glucose output and resulting in hyperglycemia (Chandrasekaran and Weiskirchen, 2024; Lu et al., 2024). In lipid metabolism, insulin typically reduces lipolysis and the release of free fatty acids (FFAs) by inhibiting hormone-sensitive lipase (HSL) and promoting lipoprotein lipase (LPL) (Zimmermann et al., 2004; Althaher, 2022). However, under IR conditions, these regulatory mechanisms are impaired, leading to elevated plasma FFAs, promoting the liver to synthesize VLDL, increase triglycerides, and decrease HDL-C levels (Bjornstad and Eckel, 2018). Elevated FFAs can also activate Toll-like receptor 4 (TLR4), triggering subsequent inflammatory responses (Zhang et al., 2018).

### 2.2 Inflammatory responses

Insulin resistance is often accompanied by chronic low-grade inflammation, which can be attributed to immune activation induced by metabolic abnormalities (Gilani et al., 2026). Hyperglycemia promotes the formation of advanced glycation end-products (AGEs), which activate nuclear factor-kappa B (NF-κB) signaling through their receptor RAGE, driving the expression of pro-inflammatory cytokines such as TNF-α, IL-6, and Monocyte Chemoattractant Protein-1 (MCP-1) (Hellwig et al., 2025). Simultaneously, elevated FFAs activate TLR4 and the NLRP3 inflammasome, further amplifying inflammatory signals (Poznyak et al., 2020). These pro-inflammatory cytokines can interfere with insulin signaling, as TNF-α, for example, promotes serine phosphorylation of IRS-1, blocking the PI3K/Akt pathway, thus establishing a positive feedback loop of inflammation and insulin resistance (Zhang et al., 2018; Liu J. et al., 2025).

### 2.3 Oxidative stress

Under IR conditions, sustained hyperglycemia and lipid overload lead to mitochondrial overoxidation, generating large amounts of reactive oxygen species (ROS) (Wang and Zhang, 2024). ROS oxidize LDL to form oxidized LDL (ox-LDL), which is taken up by macrophages via receptors like CD36 and LOX-1, promoting foam cell formation, a key step in atherosclerosis (Poznyak et al., 2021). Additionally, ROS can directly damage endothelial cells, induce apoptosis, and activate inflammation-related signaling pathways such as MAPK and NF-κB, exacerbating inflammation and cellular stress (Hong et al., 2024).

### 2.4 Endothelial dysfunction

IR impairs the PI3K/Akt/eNOS signaling pathway, reducing nitric oxide (NO) production and weakening vasodilation (Saadati et al., 2025). At the same time, high ROS and ox-LDL reduce NO bioavailability, further aggravating endothelial dysfunction. On the other hand, activation of the MAPK pathway in IR enhances the expression of angiotensin II and endothelin-1, inducing vasoconstriction and vascular remodeling (De Nigris et al., 2015; Dt et al., 2017). Furthermore, the pro-inflammatory state induced by IR activates endothelial cell expression of adhesion molecules such as intercellular adhesion molecule-1 (ICAM-1) and vascular cell adhesion molecule-1 (VCAM-1), promoting monocyte adhesion and migration, which ultimately leads to chronic vascular inflammation and atherosclerosis (Zheng et al., 2022).

### 2.5 Renal dysfunction

IR causes kidney damage through multiple pathways. First, hyperglycemia enhance oxidative stress and inflammatory responses in glomerular mesangial cells and renal tubular epithelial cells, promoting cell apoptosis and fibrosis (Jin et al., 2023). Second, the activation of the renin-angiotensin-aldosterone system (RAAS) under IR conditions leads to vasoconstriction, glomerular hyperperfusion, and hyperfiltration, further exacerbating renal injury (Feng et al., 2025). Additionally, lipotoxicity through renal lipid deposition induces tubulointerstitial injury (Rinaldi et al., 2022). Particularly in obesity-related IR, disruption of the glomerular filtration barrier structure and increased urinary albumin excretion are important precursors to the progression of chronic kidney disease (CKD) (Romagnani et al., 2025).

### 2.6 Metabolic-inflammatory-cardiorenal interaction axis

The core of CKM Syndrome is the pathological interaction of multiple organ systems under the backdrop of metabolic abnormalities. IR not only initiates metabolic dysregulation but also drives multi-organ damage through a cascade of inflammation, oxidative stress, endothelial dysfunction, and organ injury across the heart, kidneys, and vasculature, forming a positive feedback mechanism of multi-system damage (Lonardo and Weiskirchen, 2025). Importantly, these mechanisms do not occur in isolation but exhibit synergistic comorbid characteristics. For instance, elevated FFAs activate inflammation and promote renal tubular injury, while also inducing myocardial lipotoxicity and structural remodeling; meanwhile, renal dysfunction, through the accumulation of uremic toxins, promotes inflammation and vascular damage, increasing the burden on the heart (Zhang et al., 2022; Nishi et al., 2019; Lekawanvijit et al., 2012).

## 3 Novel biomarkers for insulin resistance

### 3.1 TyG index

The TyG index, a reliable and simple marker for assessing IR, has shown significant potential in risk prediction. This index integrates fasting blood glucose and serum triglycerides to reflect the lipid-glucose metabolic dysregulation driven by IR. Its formula is given by: TyG Index = Ln [fasting triglycerides (mg/dL) × fasting blood glucose (mg/dL)/2] (Ramdas et al., 2022). Numerous studies have shown that the TyG index is associated with the incidence and mortality of CKM syndrome. For example, a prospective cohort study demonstrated that the TyG index independently predicts all-cause mortality (HR = 1.39, 95% CI 1.07–1.79) and cardiovascular mortality in patients with type 2 diabetes (Sbriscia et al., 2025). It is also independently associated with major adverse cardiovascular events, diabetic nephropathy, and neuropathy. A study by Qin Zhang et al. found a U-shaped association between the TyG index and all-cause mortality in American cardiovascular patients with diabetes or prediabetes (Zhang et al., 2023). The underlying mechanism of this non-linear pattern remains to be fully elucidated. One possible explanation is that very low TyG levels in certain individuals may reflect frailty, malnutrition, advanced systemic disease, or intensive metabolic treatment, thereby introducing reverse causality or residual confounding. In contrast, elevated TyG levels are more directly associated with glucotoxicity, lipotoxicity, and heightened metabolic stress. Differences in baseline disease severity, diabetes duration, and population heterogeneity may further contribute to variation in risk-shape patterns across studies. In a multicenter study involving 959 participants, the TyG index was shown to predict the risk of cardiovascular events in patients with end-stage renal disease and coronary artery disease (CAD) (Xie et al., 2023). Menghe Wang et al. demonstrated that the TyG index could independently predict mortality, with a stronger association observed in CKM Syndrome stage 3 (Wang et al., 2025). A nationwide longitudinal survey involving 7,364 participants also confirmed these findings (Hong et al., 2025).

### 3.2 TyG derivative indices

To further enhance predictive efficacy, researchers have developed derivative indices based on the TyG index, including the dynamic TyG index and the combined TyG index. The dynamic TyG index monitors changes in the TyG index over time, reflecting the metabolic status of individuals. Studies show that baseline measurements of the TyG index alone do not fully capture its longitudinal association with outcomes, and continuous monitoring of metabolic indicators over time is needed for more accurate assessments (Lee et al., 2024). Moreover, various methods have been proposed to evaluate TyG index variations, such as calculating the individual TyG index’s pre- and post-change, cumulative TyG index, average TyG index, and using latent class trajectory models to analyze dynamic changes. For example, Cui et al. introduced the cumulative TyG index (average TyG value × continuous monitoring duration), which quantifies long-term metabolic burden and has a significant dose-response relationship with cardiovascular disease (CVD) risk (Cui et al., 2022). A prospective cohort study involving 56,149 participants found that higher TyG trajectory was significantly associated with an increased risk of heart failure (Zheng et al., 2024).

The combined TyG index enhances the predictive capacity of the TyG index by integrating various physiological and metabolic parameters. This combined approach not only considers fasting glucose and triglycerides but also includes other factors such as body composition indices, inflammatory markers, or lipid profiles, providing a more comprehensive reflection of the multidimensional features of insulin resistance.

As essential indicators for assessing metabolic status, anthropometric indices are commonly referenced in clinical practice, with body mass index (BMI) serving as a primary parameter. A prospective cohort study involving 7,376 participants focused on patients with CKM syndrome stages 0–3, revealing a significant linear positive correlation between the TyG-BMI index and cardiovascular disease (CVD) incidence (overall P < 0.001; non-linearity test P = 0.355). Specifically, for every 10-unit increase in the TyG-BMI index, the risk of CVD development elevated by 6.5% (95% confidence interval: 1.041–1.090) (Li et al., 2024). Additionally, Wen Pan et al.'s identified that in critically ill patients with stage 4 CKM syndrome, each standard deviation increase in the TyG-BMI index was associated with a 17% reduction in all-cause mortality within 180 days (hazard ratio [HR] = 0.83, 95% CI: 0.76–0.91) and a 21% decrease in 1-year all-cause mortality (HR = 0.79, 95% CI: 0.71–0.87) (Pan et al., 2025). However, given that BMI cannot accurately reflect fat distribution and is susceptible to confounding factors, researchers have employed more precise anthropometric indices to evaluate patients’ metabolic conditions, such as waist circumference (WC), waist-to-hip ratio (WHpR), and TyG-WHtR (waist-to-height ratio) (Li et al., 2025).

Systemic inflammation represents a common pathological feature in the pathogenesis of cardiovascular disease. Numerous studies have demonstrated that high-sensitivity C-reactive protein (hsCRP) not only serves as a reliable clinical biomarker for inflammatory responses but also plays a significant role in cardiovascular risk stratification (Santas et al., 2024). Based on this evidence, researchers have attempted to combine hsCRP with the TyG index to identify high-risk individuals for cardiovascular disease from the perspective of insulin resistance-inflammation interplay. A study based on the China Health and Retirement Longitudinal Study (CHARLS) revealed the important value of this combined indicator. The findings indicated that co-exposure to elevated TyG index and hsCRP levels was closely associated with a significant increase in cardiovascular disease event risk (Feng et al., 2023). Furthermore, inflammation acted as a notable mediator in the relationship between insulin resistance and cardiovascular risk (Cui et al., 2024). These results align with a review article’s conclusion that pro-inflammatory phenotypes contribute significantly to the association between cardiovascular disease and insulin resistance (Aroor et al., 2013).

Finally, several studies have explored the potential of other TyG-derived indices for risk stratification. A retrospective study revealed that both the TyG index and non-HDL-C are independent risk factors for coronary heart disease development, and their combination can better predict coronary heart disease occurrence, with an area under the ROC curve of 0.724 (95% CI: 0.681–0.768) (Zhang et al., 2025). Wu X et al.'s demonstrated that the TyG index and remnant cholesterol (RC) are independent, complementary, and easily accessible biomarkers for coronary artery disease risk assessment (Wu et al., 2025). Similarly, A retrospective study also indicated that the TyG index and RC are significantly associated with coronary artery disease risk (Table 2) (Wu et al., 2024).

**TABLE 2:** Summary of key studies on the TyG index and its derivatives in CKM Syndrome-related outcomes.

Index	Population	Study population	Main findings	Notes
TyG index (Sbriscia et al., 2025)	n = 568	Patients with type 2 diabetes (T2D)	All-cause mortality: HR 1.39 per SD increase (95% CI: 1.07–1.79)	Strongest association observed in individuals aged 57–74 years
TyG index (Zhang et al., 2023)	n = 1,072 (NHANES)	CVD patients with diabetes or prediabetes	All-cause: HR 0.47 (0.27–0.81) when TyG <9.05; HR 1.42 (1.02–1.99) when TyG >9.05. CVD death threshold 8.84; above threshold HR 1.77 (1.08–2.91)	U-shaped association with mortality; below threshold protective, above threshold harmful
TyG/TyG-BMI/WC/WHtR (Wang et al., 2025)	n = 463,545	Older adults with CKM stage 0–3 (GOLD-Health cohort)	Q4 vs Q1 HR: TyG 1.150; TyG-BMI 1.116; TyG-WC 1.190; TyG-WHtR 1.177 (all P ≤ 0.001)	TyG-WC showed highest predictive performance (AUC 0.763); stronger associations observed in CKM stage 3
TyG-Traj * (Li et al., 2024)	n = 56,149	Community-dwelling adults with 3 repeated TyG measurements (2006–2010), free of HF and cancer at baseline	Elevated-stable vs low-stable: HR 1.55 (95% CI: 1.15–2.08) for incident HF; moderate high-stable: HR 1.32 (1.08–1.60); moderate low-stable: HR 1.17 (0.99–1.37)	Four trajectories identified (low-stable, moderate low-stable, moderate high-stable, elevated-stable); association independent of baseline TyG; stronger effect in ≥60 years age group
TyG-WC (Li et al., 2025)	n = 9,432 (NHANES)	Hypertensive adults aged ≥20 years	Adjusted HR 1.002 per unit increase (95% CI: 1.001–1.002; P < 0.001)	TyG-related indices showed L-shaped relationships with all-cause and cardiovascular mortality
TyG-WHtR (Li et al., 2025)	n = 9,432 (NHANES)	Hypertensive adults aged ≥20 years	Per 1-unit increase: all-cause mortality HR 1.417 (P < 0.001); Q4 vs Q1: HR 1.677 (95% CI: 1.288–2.184)	Strongest association among TyG derivatives
TyG + hsCRP (Feng et al., 2023)	n = 9,626	Nationally representative cohort of Chinese middle-aged and older adults	High hsCRP/high TyG vs low/low: HR 1.17 (95% CI: 1.03–1.37)	Linear associations observed with incident CVD.
TyG + non-HDL-C (Wu et al., 2025)	n = 495	Patients with suspected CAD undergoing coronary angiography	AUC 0.719 (95% CI: 0.675–0.763) for TyG; AUC 0.652 (95% CI: 0.605–0.700) for non-HDL-C	Combined assessment improved diagnostic performance

* Triglyceride-Glucose Index Trajectory.

## 4 Limitations of current evidence

Despite the promising potential of the TyG index and its derivatives for prognostic evaluation in CKM Syndrome, several key limitations remain, hindering their widespread implementation in clinical practice (Tao et al., 2022). First, there is no consensus on a unified risk stratification threshold for the TyG and related indices, as different studies employ varying cutoff values, affecting the comparability of results and consistency of clinical interpretations (Alotaibi et al., 2025). Second, most current data come from specific populations, and the effects of race, gender, age, obesity, and comorbid conditions (such as diabetes and chronic kidney disease) on predictive efficacy have not been fully elucidated, limiting the generalizability to diverse clinical settings (Nayak et al., 2024; Yang et al., 2025). Moreover, some studies suggest a non-linear relationship between the TyG index and CKM Syndrome -related outcomes, such as U-shaped or J-shaped risk curves, indicating complex biological implications at different risk levels, but the mechanisms behind this are not well understood (Zhang et al., 2023; Zhao et al., 2025). Regarding dynamic applications, although dynamic TyG provides a more comprehensive reflection of metabolic risk changes, there are no standard protocols for monitoring frequency, time intervals, or data integration methods, and large-scale implementation faces challenges related to cost and technology (Nayak et al., 2024). Lastly, while the combined TyG index model may improve predictive ability, its complexity and lack of simplified, standardized clinical tools pose practical challenges, especially in resource-limited healthcare settings.

## 5 Future perspectives

To promote the clinical translation of the TyG index and its derivatives in CKM Syndrome risk management, future research should explore several dimensions. Mechanistically, system biology and multi-omics technologies (such as transcriptomics, metabolomics, and epigenomics) should be leveraged to further elucidate the molecular mechanisms by which insulin resistance mediates cardiovascular and renal injury through inflammation, lipotoxicity, and oxidative stress, strengthening the biological rationale for the biomarkers and identifying potential therapeutic targets. Large-scale, multi-center, cross-population prospective cohort studies are urgently needed to validate the generalizability and stability of the TyG index across different racial, disease spectra, and clinical scenarios, providing high-quality evidence for standardization. Additionally, individualized monitoring and intervention models based on dynamic changes in the TyG index should be developed, incorporating wearable devices and mobile health platforms for continuous risk assessment and evaluating their predictive value for responses to intervention strategies such as lifestyle changes or pharmacotherapy. Machine learning and other technologies can be utilized to optimize feature selection and structural simplification in the combined index model, improving clinical feasibility while maintaining predictive efficacy. Finally, special attention should be paid to high-risk subgroups, such as those with obesity, diabetes, or CKD, to conduct precision stratification studies and develop more targeted assessment tools and management strategies, facilitating the integration of TyG indicators into precision medicine frameworks.

## 6 Conclusion

Insulin resistance is a central driver of Cardiovascular–Kidney–Metabolic Syndrome (CKM Syndrome), linking metabolic disturbances to progressive cardiovascular and renal injury. As a practical surrogate of insulin resistance, the TyG index and its derivative indices have shown increasing value for prognostic evaluation across the CKM continuum. Current evidence supports independent associations of TyG-related markers with major clinical outcomes, including cardiovascular events, kidney function decline, and all-cause mortality, highlighting their potential utility for early risk identification and risk stratification using routinely available laboratory parameters.

However, several challenges must be addressed before widespread clinical implementation. These include the lack of standardized cut-off thresholds, population heterogeneity across age, sex, obesity, diabetes, and kidney dysfunction, and the presence of non-linear associations (e.g., U-shaped or J-shaped relationships) in certain settings. In addition, although dynamic TyG trajectories and combined TyG models may enhance predictive performance by capturing long-term metabolic burden and multidimensional risk features, their monitoring protocols, analytical approaches, and clinical feasibility remain insufficiently standardized.

Future studies should prioritize multicenter prospective validation across diverse populations, establish clinically actionable thresholds, and clarify the biological mechanisms underlying non-linear risk patterns. Integration of digital health tools and machine learning approaches may further enable individualized monitoring and simplified composite models with improved clinical usability. Overall, TyG-related biomarkers represent a scalable and cost-effective strategy for earlier detection and precision risk assessment in CKM, with the potential to support timely prevention and improve long-term outcomes.
